# Computational Modeling of Flow in an in Vitro Cerebrovascular Model Under Pulsatile Conditions with Experimental Validation

**DOI:** 10.1007/s13239-025-00813-x

**Published:** 2025-12-16

**Authors:** Boyang Su, Brent A. Craven, Cody J. Kubicki, Daniel Khalil, Scott D. Simon, Keefe B. Manning

**Affiliations:** 1 Department of Biomedical Engineering, The Pennsylvania State University, University Park, PA USA; 2 Department of Mechanical Engineering, Baylor University, Waco, TX USA; 3 Department of Neurosurgery, Penn State Hershey Medical Center, Hershey, PA USA; 4 Department of Surgery, Penn State College of Medicine, Hershey, PA USA

**Keywords:** Acute ischemic stroke, Cerebrovascular CFD, Image-based modeling, Embolus migration

## Abstract

**Purpose:**

Computational fluid dynamics (CFD) has been widely used to understand various cardiovascular diseases such as acute ischemic stroke (AIS), which occurs when a blood clot lodges in the cerebrovasculature and obstructs blood flow that may lead to brain damage or death. Compared with medical imaging, CFD can predict hemodynamics and clot migration, which are crucial in better understanding the biomechanics of AIS. To rely on computational modeling, however, the simulations need to be validated by comparing with experiments

**Methods:**

In this study, we develop an in vitro experimental model of pulsatile flow in the aorta and cerebrovasculature. The model was filled with a blood analog fluid and pulsatile flow was driven by a piston pump to generate realistic physiological flow conditions. Experimental measurements of the time-varying pressure and flow rate were acquired and are used to validate corresponding CFD simulations

**Results:**

CFD predictions of the time-averaged pressure at the outlets are shown to be within 8% of the experimental measurements, while the time-averaged flow rate is within 1%.

**Conclusions:**

This work demonstrates a promising capability for modeling embolus migration and lodging in the brain. Future work will validate simulations of clot migration that may be used to better understand AIS biomechanics and treatment options.

## Introduction

Acute ischemic stroke (AIS) occurs when an embolus migrates to the brain and blocks blood flow, requiring immediate intervention to prevent long-term disability or death. In a healthy adult, the cerebral blood flow rate is typically around 750 ml/min, accounting for approximately 16% of the cardiac output [1]. The brain receives its blood supply through an intricate network of arteries, primarily the carotid and vertebral arteries, which branch into smaller vessels within the brain. This vascular network is vital for delivering oxygen, glucose, and other essential nutrients throughout the brain. The urgency of promptly treating AIS cannot be overstated, as prolonged deprivation of oxygen and nutrients significantly increases the risk of irreversible brain damage [2].

Most ischemic strokes are embolic in nature [3, 4] and are caused by formed blood clots in the upstream circulatory system (e.g., the carotid artery or heart) that embolize and migrate to the brain. These clots often obstruct the anterior, middle, or posterior cerebral arteries or one of their branches. Embolic strokes are frequently associated with atrial fibrillation, a condition that promotes clot formation in the heart [5]. Additional risk factors include heart valve disease and certain autoimmune disorders [6, 7]. AIS is usually treated pharmacologically through the use of thrombolytics to dissolve the clot, via mechanical thrombectomy to remove it, or a combination thereof [7].

Recent work by Bhardwaj et al. [8] developed an in vitro experimental model of the cerebrovasculature and a corresponding CFD model to study AIS. The cerebrovascular model is based on representative patient anatomy and includes the aorta and the cerebral arteries. Bhardwaj and colleagues [8] acquired pressure and flow rate measurements in the model under normal and stroke conditions. Corresponding CFD simulations were also performed that closely matched the experiments. The study, however, only considered steady flow conditions, whereas physiological flow in the cerebrovasculature is pulsatile in nature [9–13].

The objective of this study is to extend the work of Bhardwaj et al. [8] by considering pulsatile flow conditions. We first perform experiments in the in vitro cerebrovascular model, measuring the time-varying pressure and flow rate under realistic pulsatile conditions. CFD simulations are then performed and compared with the experimental measurements for validation. This research lays the foundation for future work on studying clot migration and lodging to better understand the biomechanics of AIS.

## Methods

### In vitro Experiments

**Fig. 1:**
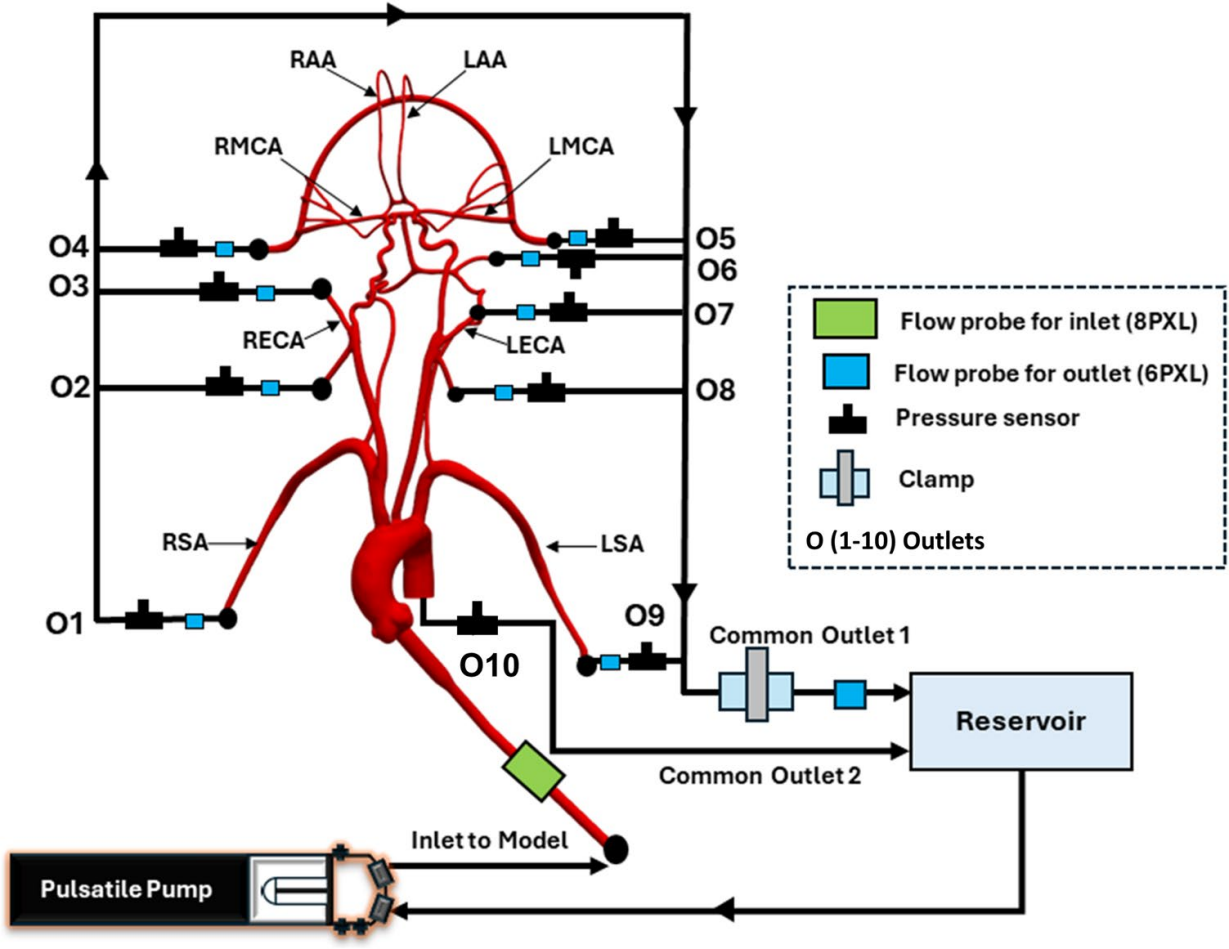
Schematic of the in vitro circulatory flow loop used for pulsatile flow rate and pressure measurements

This study used the realistic aorta and cerebrovascular model developed by Bhardwaj et al. [8] that is based on representative patient anatomy. As illustrated in Fig. 1, the in vitro silicone model consists of one inlet to the aorta and ten outlets. To conduct experiments, a mock circulatory loop was used consisting of a pulsatile pump (ViVitro Labs, Inc., Victoria, BC, Canada), a fluid reservoir, a flow meter (T402, Transonic Systems, Inc., Millis, MA), ultrasonic flow probes (8PXL and 6PXL, Transonic Systems, Inc., Millis, MA), and pressure transducers (Millar pressure catheter, Millar, Inc.; DPT-100, Deltran, Utah Medical Products, Midvale, UT). Each component location is shown in Fig. 1. A multi-channel data acquisition system (516 E, IOtech) was used for data collection. A Newtonian blood analog comprised of 60% water and 40% glycerol (by weight) was used as the working fluid. The fluid was the same as in [8] with a density of 1.09 ± 0.03 g/ml and dynamic viscosity of 3.98 ± 0.14 cP. Pulsatile flow experiments were carried out over six cardiac cycles. The cardiac rate was 71 beats/min (bpm) with an average inlet flow rate of 4.91 L/min, roughly equivalent to the cardiac output of a healthy adult.

Experiments were conducted at 22.2 °C to measure the time-varying pressure and flow rate in the model. Similar to Bhardwaj et al. [8], to obtain a realistic flow split in the model, we adjusted the clamp at Common Outlet 1 (Fig. 1) to obtain a flow distribution with approximately 76% of the time-averaged flow passing through the descending aorta and the remaining 24% of the flow passing through the arteries originating from the aortic arch.

## CFD Simulation of Flow

**Fig. 2:**
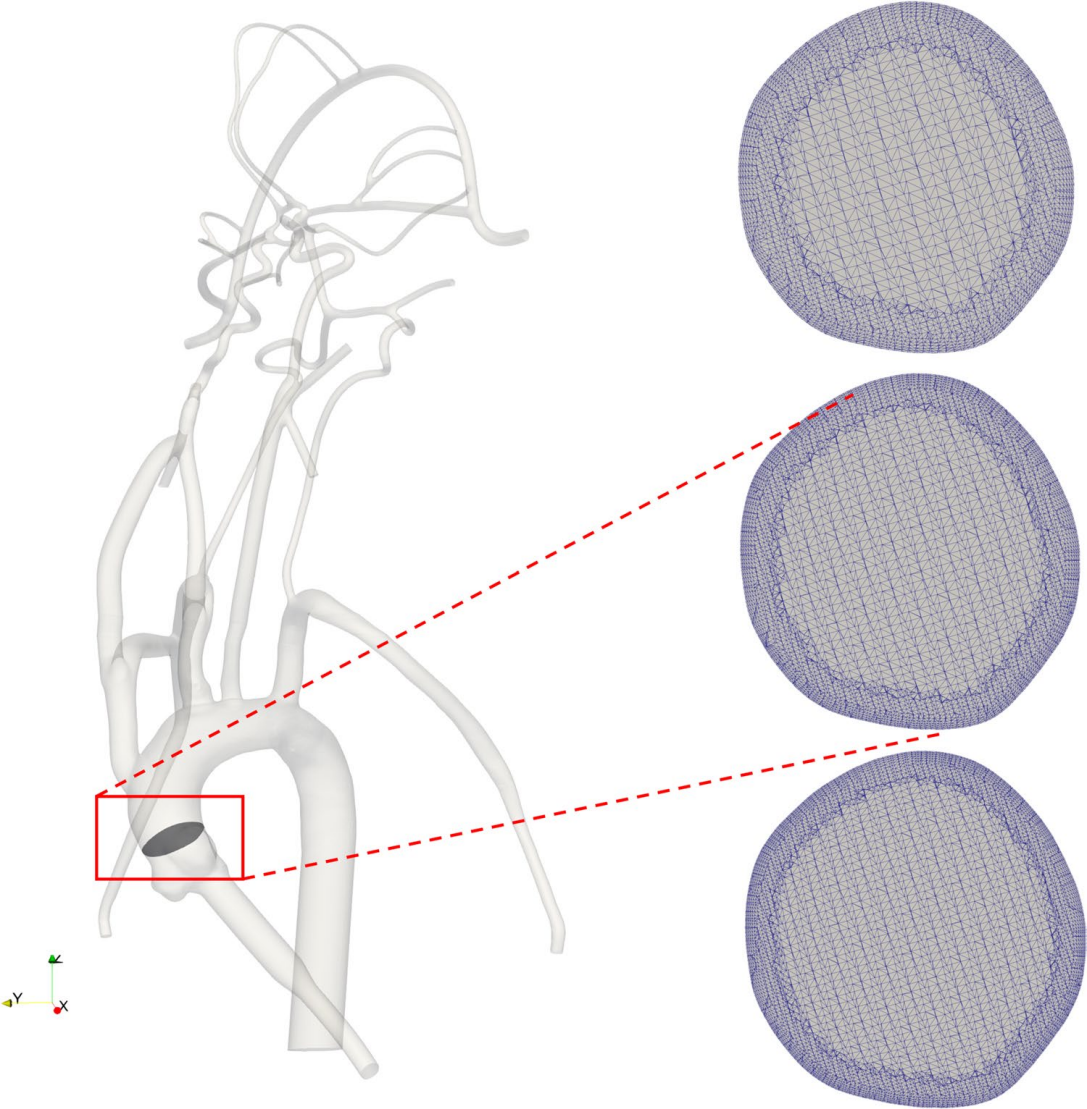
**A** Illustration of the CFD cerebrovascular model with a cut plane in the aortic arch showing a cross-section of the **B** coarse, **C** medium, and **D** fine mesh

As illustrated in Fig. 2, three unstructured hexahedral meshes were created using the commercial software CF-MESH + (version 4.5; Creative Fields, Croatia). The three meshes (coarse, medium, and fine) contained approximately 3.3 million, 6.6 million, and 10.7 million computational cells, respectively, with five wall-normal layers to resolve the near-wall velocity gradient. As summarized in Table 1, a CFD simulation was performed with each of the three meshes to ensure that the results are insensitive to the mesh resolution.

**Table 1:** The time-averaged pressure at each outlet from the CFD mesh refinement study

Outlets	(grid size in million)				
	3.41	(Diff %)	6.55	(Diff %)	10.72
*Pressure at each outlet (mm Hg)*					
Outlet 1	84.84	0.28	84.55	0.07	84.61
Outlet 2	83.37	0.31	83.04	0.08	83.11
Outlet 3	83.79	0.32	83.47	0.07	83.53
Outlet 4	82.31	0.35	81.97	0.07	82.03
Outlet 5	81.38	0.34	81.04	0.07	81.10
Outlet 6	83.65	0.34	83.36	0.00	83.36
Outlet 7	84.38	0.38	83.96	0.12	84.06
Outlet 8	82.07	0.42	81.65	0.10	81.73
Outlet 9	83.90	0.22	83.77	0.07	83.71
Outlet 10	84.96	0.00	84.94	0.01	84.96

CFD simulations of incompressible flow were performed using OpenFOAM (version 2.31) with the k-ω shear stress transport (SST) turbulence model [14, 15]. Specifically, the simulations used OpenFOAM’s hybrid PISO-SIMPLE (PIMPLE) solver, pimpleFoam, with second-order accurate spatial and temporal discretization schemes. The simulations were performed for a total elapsed time of approximately 3.4 seconds (4 cardiac cycles) to overcome the transient flow initialization and to obtain a pulsatile periodic steady-state condition. Time-averaged results were computed using the last cardiac cycle. The raw CFD simulation results were post-processed and visualized using ParaView (Kitware, Inc., Clifton Park, NY).

Pulsatile boundary conditions were applied based on the in vitro experiments. This was accomplished using a two-staged approach. First, an initial simulation was performed by prescribing the measured time-varying flow rates at the aortic inlet and the outlets of the left subclavian artery (LSA), right subclavian artery (RSA), and the cerebral arteries (outlets 1–9 in Fig. 1). A pulsatile pressure outlet condition was assigned for the aortic outlet based on the experiments (outlet 10 in Fig. 1). This initial simulation yielded the pressure waveforms corresponding to the measured flow rates through outlets 1–9. In the second step, the time-varying pressures from outlets 1–9 were extracted from the initial simulation and applied as pulsatile outlet pressure boundary conditions along with the measured pressure profile at the aortic outlet and the measured flow rate waveform at the aortic inlet. OpenFOAM’s *pressureInletOutletVelocity* boundary condition was used for the velocity at all pressure outlets, which assigns a zero-gradient velocity condition on boundary patch faces with outflow and an extrapolated fixed-value (i.e., Dirichlet) condition for velocity on patch faces that have reversed inflow [8].

## Results

### In Vitro Experiments

**Fig. 3:**
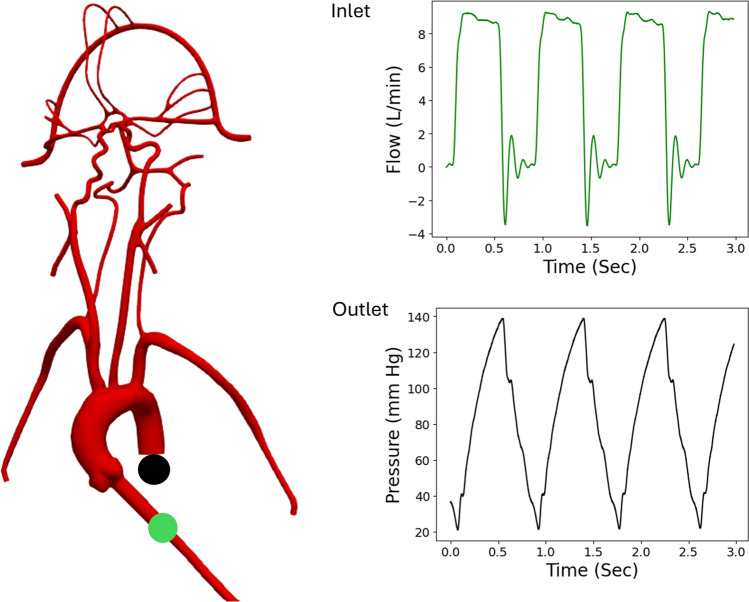
Boundary conditions for the CFD simulations illustrating the time-varying flow rate prescribed at the aortic inlet and an example of the pressure waveform applied at the outlet of the descending aorta based on the in vitro experimental measurements

**Fig. 4:**
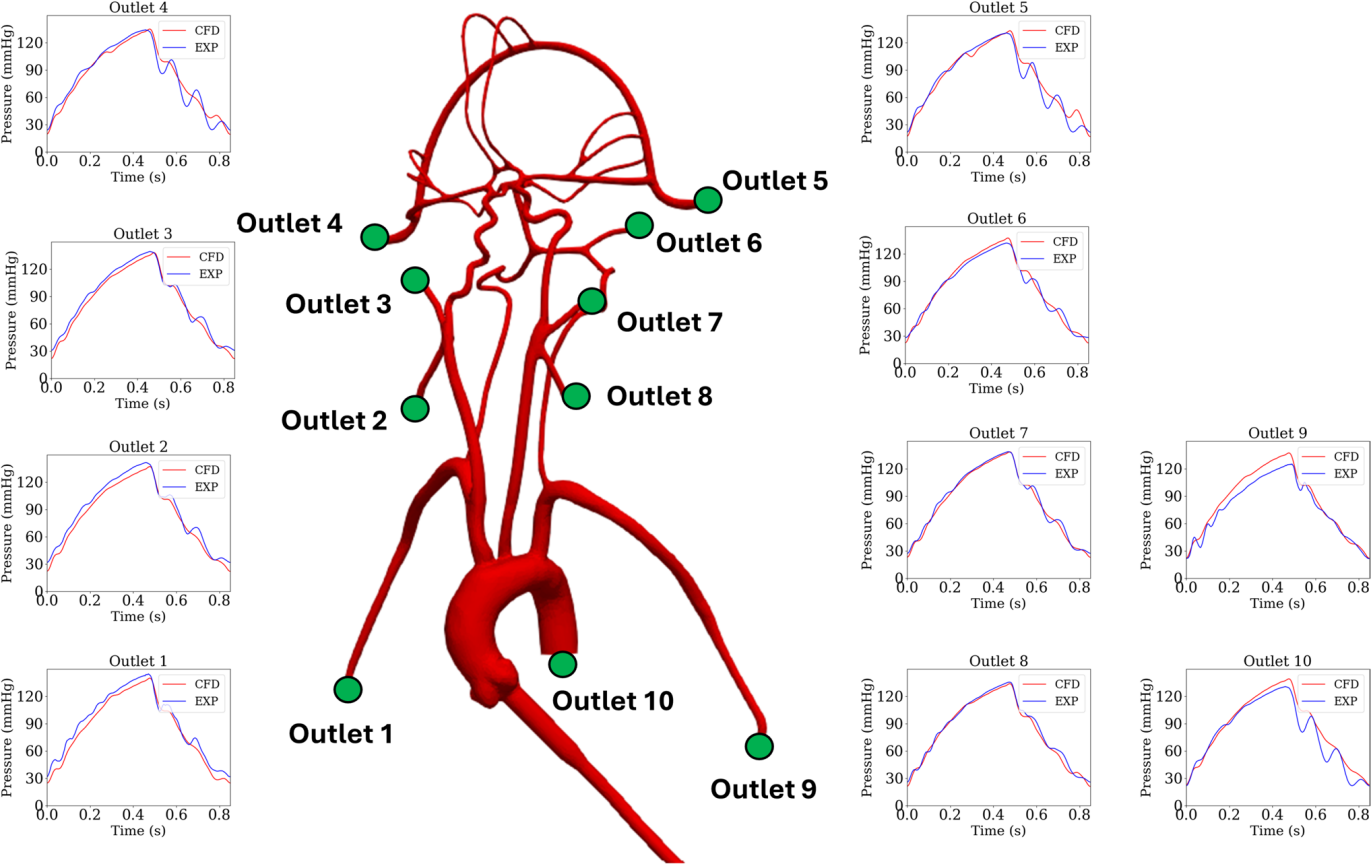
Comparison of pulsatile pressure waveforms from in vitro experiments and CFD

Figure 4 shows the pulsatile pressure waveforms measured in the Outlets 1-9 in the in vitro model. The pressure waveforms are similar in each of these outlets. The measured flow split in the model consisted of approximately 76% of the time-averaged flow passing through the descending aorta with the remaining 24% flowing through the arteries originating from the aortic arch. The time-varying velocity profile at the aortic inlet is representative of a physiological waveform, consisting of both systole and diastole as shown in Fig. 3.

## CFD Simulations

Figure 4 also shows the comparison of the computed pressure waveforms from CFD with the experimental measurements at each outlet under pulsatile flow conditions. Generally, the pressure waveforms from CFD and the experiments show relatively good agreement. Table 2 summarizes the quantitative comparison of the time-averaged pressure and flow rate at each outlet. The time-averaged pressures from CFD agree relatively well with the experiments, most to within 5% (Table 2). The comparison of time-averaged flow rate through each outlet is excellent, with the largest percent difference of less than 1% occurring at Outlet 10 (the descending aorta).

**Table 2:** Comparison of the percent difference between experiments and CFD for the time-averaged flow rate and pressure through each outlet

Location	Flow rate (%)			Pressure (mm Hg)		
	CFD	EXP	Diff	CFD	EXP	Diff (%)
Outlet 1	2.16	2.04	0.13	84.42	90.67	6.90
Outlet 2	2.98	3.06	0.08	82.92	88.67	6.48
Outlet 3	3.33	3.47	0.14	83.35	87.11	4.32
Outlet 4	0.94	0.79	0.15	81.85	82.19	0.41
Outlet 5	3.66	3.75	0.08	80.93	79.38	1.94
Outlet 6	0.48	0.43	0.05	83.24	82.28	1.16
Outlet 7	0.42	0.50	0.08	83.92	85.65	2.03
Outlet 8	4.95	5.23	0.28	81.52	83.69	2.60
Outlet 9	4.47	4.85	0.38	83.65	77.66	7.71
Outlet 10	76.6	75.87	0.73	84.86	84.79	0.08

**Fig. 5:**
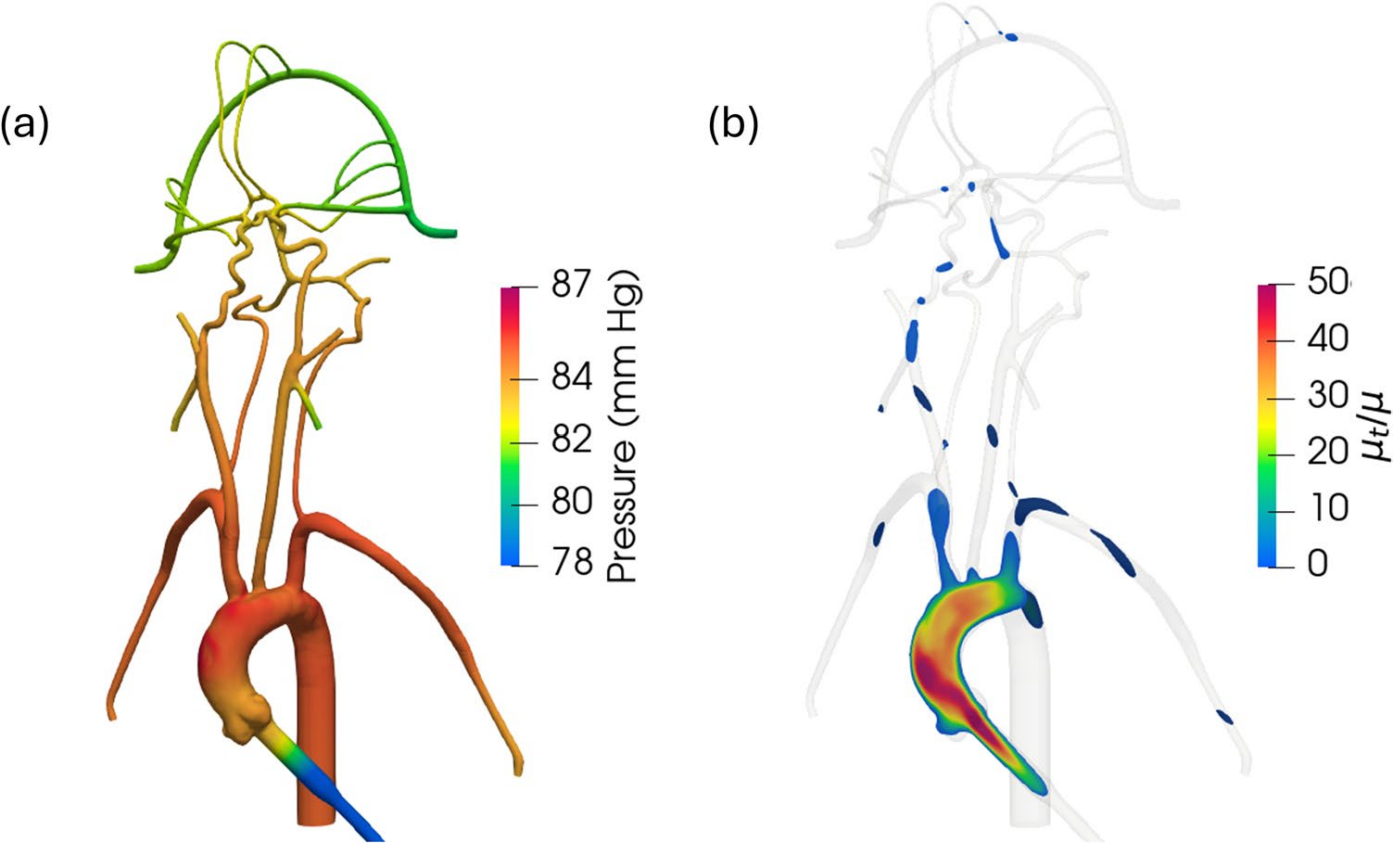
**a** Contours of surface pressure in the cerebrovascular model. Both (a) and (b) illustrate time-averaged results over one cardiac cycle. **b** Contours of turbulent viscosity ratio, ${\mu }_{t}/\mu$, illustrating regions of laminar and turbulent flow, indicated by small and large values of ${\mu }_{t}/\mu$, respectively

Figure 5a illustrates contours of time-averaged wall pressure over one cardiac cycle from CFD. As expected, the highest pressures occur in the aorta with pressure decreasing in the cerebral arteries. As the dominant driving force, the pressure gradient ensures the forward flow direction from the heart to the brain. CFD predicts a time-averaged pressure gradient of around 6 mmHg between the aorta and the cerebral arteries. In Fig. 5b, we show contours of the ratio of turbulent viscosity to molecular viscosity (µ_t_/µ), which is useful for visualizing regions of predicted laminar and turbulent flow from CFD. Specifically, in regions where µ_t_/µ is much less than one, the turbulence model is inactive and CFD, thus, predicts laminar flow. Conversely, in regions where µ_t_/µ is much greater than one, the model predicts turbulent flow. As illustrated in Fig. 5b, the *k-ω* SST model predicts turbulence in the inlet and the aorta, while laminar flow is predicted in the cerebral arteries. These results correspond to the expected flow regime in each region.

The potential impact of AIS on cerebral blood flow was also estimated by obstructing Outlet 5 in the CFD model, assuming that an embolus lodged downstream. As summarized in Table 3, blocking Outlet 5 resulted in a higher blood supply in the cerebral arteries on the contralateral side (i.e., Outlet 4), increasing from 0.94% (normal) to 2.43% (obstructed). The other major increment of flow (> 0.5%) occurred at Outlet 8 (left bifurcation), where the flow increased from 4.95% (normal) to 6.15% (obstructed). As this location is on the same side as Outlet 5, it serves to partially compensate for the obstruction.

**Table 3:** Percentage of the total time-averaged flow rate through each outlet for the normal and obstructed (i.e., stroke) conditions obtained from CFD

Location	Flow rate (%)		
	Stroke	Normal	Diff
Outlet1	2.41	2.16	0.25
Outlet2	3.16	2.98	0.18
Outlet3	3.14	3.33	-0.19
Outlet4	2.43	0.94	1.49
Outlet5	0.00	3.66	-3.66
Outlet6	0.82	0.48	0.34
Outlet7	0.80	0.42	0.38
Outlet8	6.15	4.95	1.20
Outlet9	4.25	4.47	-0.22
Outlet10	76.83	76.6	0.23

## Discussion

The primary goal of this study is to validate CFD modeling of cerebrovascular flow using an in vitro experimental model under pulsatile conditions, aiming to establish a numerical framework for the future investigation of embolus migration and lodging in AIS. The in vitro model included the aorta, the arteries originating from the aortic arch, and the downstream cerebrovasculature. It thus allows the migration of AIS emboli from the left heart, which is only partially modeled in other studies [13, 16, 17]. Flow rate and pressure waveforms were employed for comparison between experimental measurements and numerical simulations, similar to our previous study under steady state conditions [8]. For pressure, outlet waveforms all shared a similar pattern, with values generally between 20 and 140 mmHg. Time-averaged outlet pressures were between approximately 78 and 91 mmHg. These values are beyond physiological conditions for a normal human subject, but are comparable to pressure ranges observed in other studies [16, 17]. This is likely due to the limitation of using a silicone model. Even so, the numerical model was able to simulate this more extreme condition, demonstrating its capability to model various flow conditions. As summarized in Table 2, the maximum difference between the measured and simulated pressures was less than 8% and difference in flow rate were within 1%.

The study is novel in several ways. First, we incorporate measurements of pressure, which are not typically available. In the literature, CFD simulations based on clinical data typically included flow rate only without pressure information [18, 19]. Additionally, in vitro measurement-based CFD studies of cerebrovascular flow under pulsatile conditions are scarce, and models typically do not include an anatomical aorta and the arteries originating from the aortic arch (e.g., [16, 20]). Indeed, previous research on numerical modeling of the complete aorta and cerebrovascular system is limited [8, 21], and of those studies that have been conducted most do not describe the nature of the flow. In most studies investigating flow in the cerebrovasculature alone, the flow was assumed to be laminar [18, 22, 23]. When an aneurysm is present, turbulence can be occur and is often modeled using the *k-ω* SST turbulence model [24]. Aortic flow is, however, mostly turbulent [25, 26], though it is sometimes treated as laminar (e.g., [27]). Another novel aspect of this study is that we used the *k-ω* SST turbulence model to simulate both laminar and turbulent flow regimes in the cerebrovascular model. As described in the results and illustrated in Fig. 5b, we demonstrate here that this model captures both turbulence in the aorta and laminar flow in the cerebral arteries.

Finally, we note several limitations of the study. First, the cerebrovascular model used in the CFD simulation was assumed to be rigid, while the silicone model in the in vitro experiments was compliant. Previous studies have shown that wall compliance can affect local hemodynamic parameters, but its effect on global flow rates is relatively small. For example, Jodko et al. [28] reported that the differences in flow rate were below 3% at the carotid bifurcation between rigid and compliant models. Similarly, Luisi et al. [20] found relatively good agreement between rigid CFD and particle image velocimetry measurements obtained in a compliant cerebrovascular phantom. In the aortic region, rigid-wall models have been widely used, with velocity differences of less than 10% [29, 30]. Collectively, the evidence suggests that a rigid model, despite its limitations in capturing local hemodynamic variations, offers a good approximation for global flow predictions in the current study. While this is a common assumption (e.g., see [31, 32]), in future work we plan to investigate the effect of fluid-structure interaction during pulsatile flow on local cerebrovascular hemodynamics. Additionally, we do not quantitatively validate CFD predictions of velocity by comparing with measurement of the local flow field—e.g., using particle image velocimetry (PIV) [33, 34]. Acquiring PIV measurements in the in vitro model is challenging and is a topic of future work. Finally, the working fluid in both the experiments and CFD was Newtonian, while blood behaves as a non-Newtonian fluid at low strain rates. While this is also a common assumption (e.g., see [2, 18, 35–38]), we plan to investigate the influence of non-Newtonian effects in future work.

## Conclusion

Computational modeling holds tremendous potential for better understanding the biomechanics of AIS. To rely on computational modeling, however, the simulations need to be validated by comparing with experiments. In this study, we develop an in vitro experimental model of pulsatile flow in the cerebrovasculature and perform experiments that are used to validate CFD simulations. CFD predictions of the time-averaged pressure at the outlets were shown to be within 8% of the experimental measurements, while the time-averaged flow rate was within 1%. Future work will validate simulations of clot migration that may be used to better understand AIS biomechanics and treatment options.

## Data Availability

Data can be made available to interested parties via reasonable written request to the authors.

## Code availability

Codes to generate these data can be made available to interested parties via reasonable written request to the authors.
